# Rate dependent of strength in metallic glasses at different temperatures

**DOI:** 10.1038/srep27747

**Published:** 2016-06-08

**Authors:** Y. W. Wang, X. L. Bian, S. W. Wu, I. Hussain, Y. D. Jia, J. Yi, G. Wang

**Affiliations:** 1 Laboratory for Microstructures, Institute of Materials, Shanghai University, Shanghai 200444, China

## Abstract

The correlation between the strength at the macroscale and the elastic deformation as well as shear cracking behavior at the microscale of bulk metallic glasses (BMGs) is investigated. The temperatures of 298 K and 77 K as well as the strain rate ranging from 10^−6^ s^−1^ to 10^−2^ s^−1^ are applied to the BMGs, in which the mechanical responses of the BMGs are profiled through the compression tests. The yield strength is associated with the activation of the elementary deformation unit, which is insensitive to the strain rate. The maximum compressive strength is linked to the crack propagation during shear fracture process, which is influenced by the strain rate. The cryogenic temperature of 77 K significantly improves the yield strength and the maximum compressive strength of the BMGs.

Due to extraordinarily high strength, bulk metallic glasses (BMGs) are expected to be potentially applied in structural materials. Besides high strength, a considerably good toughness also makes BMGs to be attractive^1^,^2^,^3^,^4^. The combination of high strength and good toughness will lead BMGs to be used in some extreme service conditions, such as high strain rate deformation, and low environmental temperatures etc^4^,^5^,^6^,^7^,^8^. Therefore, it is required to uncover the correlation between the strength and the fracture behaviors of BMGs under different temperatures, and loading rates. Abundant researches have been carried out to explore the influence of cryogenic temperature on the strength and the plastic behavior (serrated flow) of BMGs in the past several years^5^,^9^,^10^,^11^,^12^,^13^. The quasi-static deformation tests as well as the dynamic deformation tests of many BMGs also were performed, which suggested a negative-sensitivity of strength to strain rate^6^,^8^,^14^,^15^,^16^,^17^,^18^,^19^,^20^,^21^. Furthermore, the influences of cryogenic temperature and strain rate on the deformation behavior of BMGs were discussed in the framework of deformation kinetics^22^. In these work, shear band^9^,^23^,^24^,^25^,^26^,^27^, serrated flows^28^,^29^,^30^, activation energy of shear banding^31^ etc. have been comprehensively investigated. However, few work focus on the fracture behavior of BMGs coupled influenced by strain rates and temperatures. The fracture behavior of BMGs, such as the crack formation during the fracture process, may provide useful clue to not only understand deformation mechanism but also guide the design of BMGs with high toughness.

Accordingly, in this paper, two BMGs, i.e., Zr_52.5_Cu_17.9_Ni_14.6_Al_10_Ti_5_ (at.%) (Zr-MG) and Ce_68_Al_10_Cu_20_Co_2_ (Ce-MG), with different yield strength and toughness are selected as the model materials. The compression tests of two BMGs are performed at 298 K and 77 K, respectively, with a wide strain-rate range from 2.5 × 10^−6^ s^−1^ to 2.5 × 10^−2^ s^−1^. The coupled effects of temperatures and strain rates on the crack formation of the BMGs are elucidated based on the 3 dimensional (3D) fractogrphies and the theory of the instability of the crack tip.

## Results

**Figure 1:**
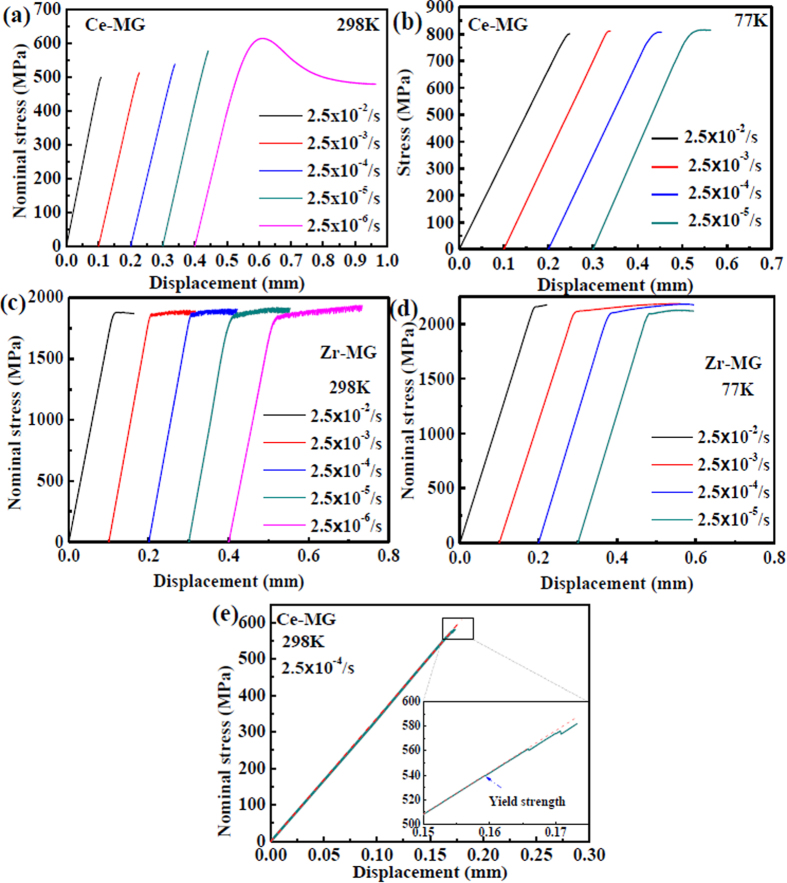
Nominal stress-displacement curves of the BMGs at different strain rates and temperatures. (**a**) Ce-MG at 298 K. (**b**) Ce-MG at 77K. (**c**) Zr-MG at 298 K. (**d**) Zr-MG at 77 K. (**e**) The determination of yield strength of the BMGs. The Ce-MG fractured at 10^−4^ s^−1^ and 298 K is representatively shown.

The compression nominal stress-displacement curves of the Zr-MG and the Ce-MG at 298 K and 77 K, and different strain rates are plotted in Fig. 1. Because the extensometer cannot be used in the cryogenic temperature, the plastic strain measurements are not provided. The results of repeated compression tests are summarized in Figs S1–S4 in Supplementary materials.

At 298 K, the deformation of the Ce-MG undergoes a remarkable brittle-to-ductile (BTD) transition with decreasing strain rate from 10^−2^ to 10^−6^ s^−1^. In the strain-rate range from 10^−5^ ~ 10^−2^ s^−1^, the stress-displacement curves of the Ce-MG exhibit a linear elastic deformation followed by a catastrophic fracture without obvious plastic flow (Fig. 1a). When the strain rate decreases to 10^−6^ s^−1^, a stress overshoot followed by a plastic flow appears (Fig. 1a). The fracture at the strain rate of 10^−6^ s^−1^ does not occur. Regarding that the glass transition temperature of the Ce-MG is 352 K^32^, the deformation temperature of 298 K is already close to the supercooled liquid region of the Ce-MG. Thus, a homogeneous deformation occurs at a relative low strain rate. When temperature comes to 77 K, the deformation of the Ce-MG in the strain-rate range from 10^−2^ to 10^−5^ s^−1^ exhibits the obviously plastic flow (Fig. 1b), and the catastrophic fracture after plastic deformation. The maximum compressive strength at 77 K is obviously larger than the value at 298 K.

For the deformation of the Zr-MG at 298 K, a linear elastic deformation followed by a plastic deformation, i.e., an elasto-plastic deformation, is observed at different strain rates (Fig. 1c). At 77 K, the elasto-plastic deformation is also observed (Fig. 1d). Furthermore, the decrease in temperature causes the maximum compressive strength to be increased, which is same to that in the Ce-MG.

**Figure 2:**
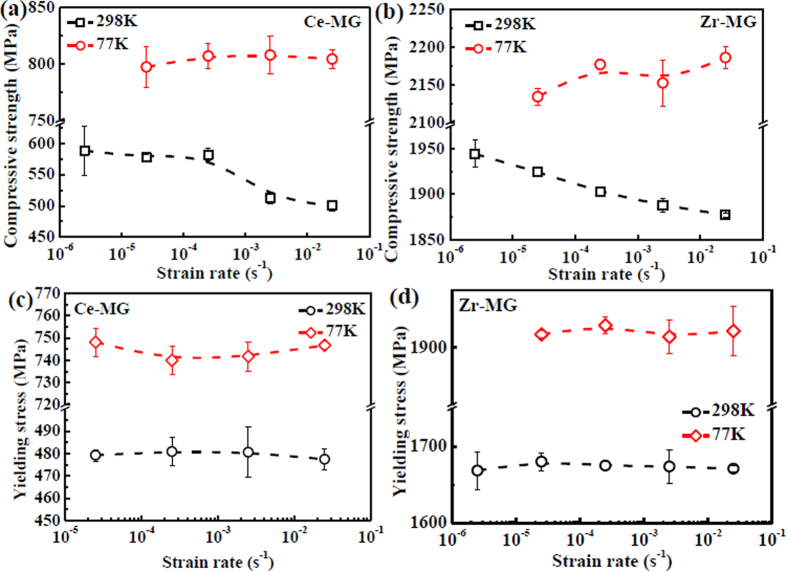
Strength of two BMGs at different strain rates and temperatures. (**a**) Maximum compressive strength of Ce-MG vs. strain rate. (**b**) Maximum compressive strength of Zr-MG vs. strain rate. (**c**) Yield strength of Ce-MG vs. strain rate. (**d**) Yield strength of Zr-MG vs. strain rate.

According to the stress-displacement curves of two BMGs, the maximum compressive strength and the yield strength are chosen to characterize the mechanical response of the BMGs at different strain rates and temperatures, which are summarized in Fig. 2. The strength values are the average values from repeated compression tests (Figs S1–S4 in Supplementary materials). It can be seen that the compressive strength of the Ce-MG at 298 K decreases from 588 ± 39 to 500 ± 9 MPa, and the value of the Zr-MG decreases from 1945 ± 15 to 1877 ± 2 MPa with increasing strain rate from 10^−5^ to 10^−2^ s^−1^ (Fig. 2a,b), which clearly suggests a negative strain-rate sensitivity. At 77 K, the strain-rate sensitivity is not obvious in both BMGs. The compressive strength of the Ce-MG remains unchanged around a stress of 805 MPa with increasing the strain rate (Fig. 2a). For the Zr-MG at 77 K, although it seems that the compressive strength increases slightly from 2134 ± 11 to 2186 ± 15 MPa with strain rate, regarding the error bars, we believe that the compressive strength is kept at a constant (Fig. 2b). The yield strength of the BMGs is determined as the elastic stress deviating the linear elastic stress-displacement curve, which is representatively shown in Fig. 1e. The yield strengths of the Ce-MG at 298 K and 77 K are almost constants of 480 ± 11 MPa and 745 ± 6 MPa, respectively (Fig. 2c), and the corresponding values of the Zr-MG are 1675 ± 24 MPa and 1910 ± 21 MPa, respectively (Fig. 2d). The change of the strain rate does not significantly influence the yield strength of the BMGs.

**Figure 3:**
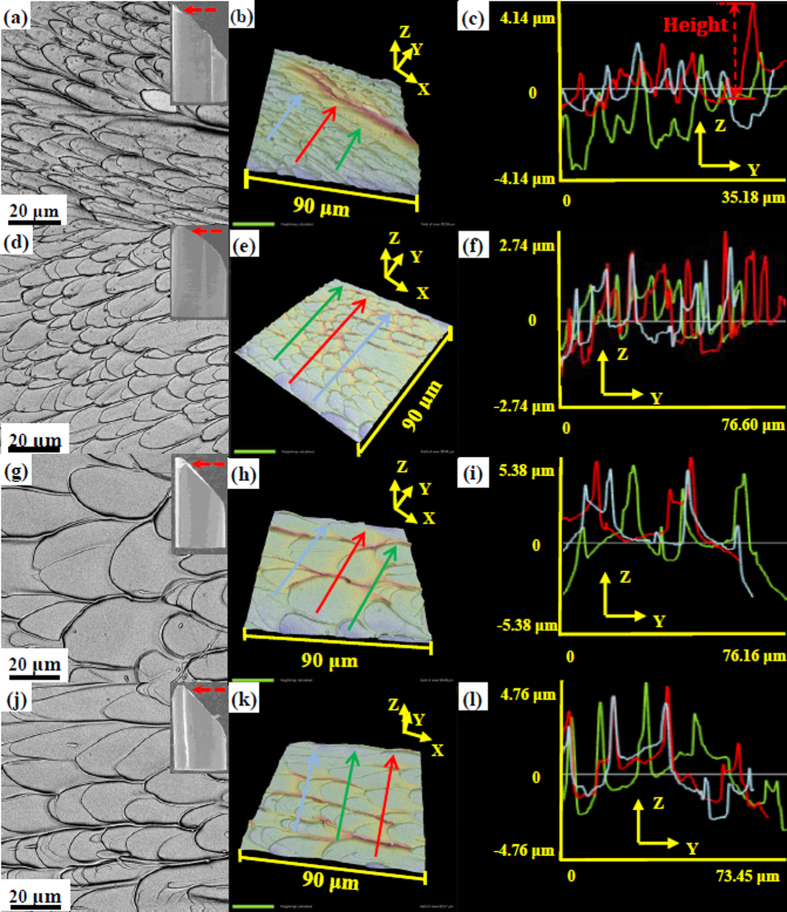
Fracture morphologies of two BMGs fractured at different temperatures with the strain rate of 2.5 × 10^−4^ s^−1^. (**a**) Fractography of Ce-MG at 298 K. (**b**) 3D fractographies of Ce-MG at 298 K. (**c**) Three sectional shapes reflecting the surface roughness along the three lines in (**b**). (**d**) Fractography of Ce-MG at 77 K. (**e**) 3D fractographies of Ce-MG at 77 K. (**f**) Three sectional shapes reflecting the surface roughness along the three lines in (**e**). (**g**) Fractography of Zr-MG at 298 K. (**h**) 3D fractographies of Zr-MG at 298 K. (**i**) Three sectional shapes reflecting the surface roughness along the three lines in (**h**). (**j**) Fractography of Zr-MG at 77 K. (**k**) 3D fractographies of Zr-MG at 77 K. (**l**) Three sectional shapes reflecting the surface roughness along the three lines in (**k**). Red dash-line arrows in the insets point to the SEM observation regions.

The fractographies of two BMGs at the strain rate of 10^−4^ s^−1^, and at different temperatures are representatively shown in Fig. 3. The BMGs fractured at other strain rates are also shown in Figs S5–S8 in Supplementary materials. Generally, cracking process in materials includes two stages, i.e., crack-formation stage and crack-propagation stage. The crack formation is associated with the intrinsic strength of materials. Therefore, the SEM observation region is located on the edge of fracture surface (which is marked by red arrows in Fig. 3), where the crack started to propagate. The SEM images of the Ce-MG and the Zr-MG (the left column of Fig. 3), show that the fracture surfaces are fully occupied by the finger-like vein patterns accompanied with some liquid droplets. The size of the vein pattern in the Zr-MG is larger than that in the Ce-MG. The insets of the left column images are the side views of the fractured BMGs, which confirm that both the Ce-MG and the Zr-MG behave a shear fracture behavior, and the shear-fracture angles of the Ce-MG and the Zr-MG are 44° and 42°, respectively. The changes in strain rate and temperature do not influence the fracture angle. The 3D fractographies are also reconstructed in the middle column of Fig. 3, which can quantitatively provide the roughness of surface morphologies. Coordinates are marked on the 3D fractographies (the middle column of Fig. 3). The crack propagates along the *X*-axis on the *X-Y* plane. Based on the 3D fractographies, the sectional shape on the *Y-Z* plane, i.e., perpendicular to the crack-propagation direction, is profiled in the right column of Fig. 3, which clearly presents the roughness (height) of the finger-like vein pattern. For each fracture surface, three sectional shapes at different positions are profiled, as shown in the middle and right columns of Fig. 3.

**Figure 4:**
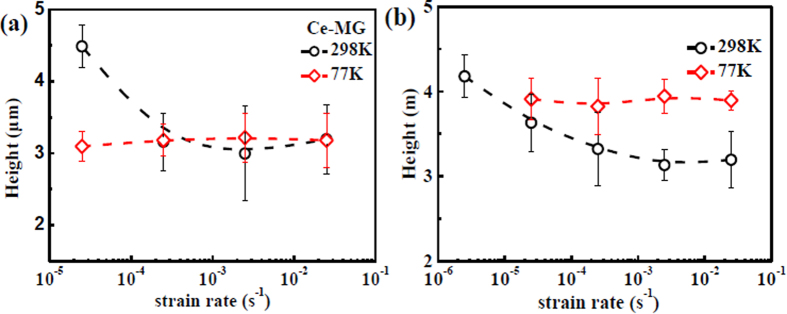
The heights of the finger-like vein patterns of two BMGs as functions of strain rate at different temperatures. (**a**) Ce-MG. (**b**) Zr-MG.

According to the sectional shape of the fracture surface (the right column of Fig. 3), the average height of the finger-like vein pattern at different strain rates and temperatures are plotted in Fig. 4. The measurement of the height of the finger-like vein pattern is schematically shown in Fig. 3c, indicating that the relative height of the peak in the sectional shape of the fracture surface is considered as the height value. At 298 K, with increasing strain rate, the heights of the finger-like vein patterns of the Ce-MG and the Zr-MG decrease from 4.5 μm to 3 μm, and from 4.2 μm to 3.2 μm, respectively. When the temperature decreases to 77 K, the heights of the finger-like patterns of the Ce-MG and the Zr-MG are almost constants of 3 μm and 4 μm, respectively, which are independent of strain rate.

## Discussion

Yield strength, as a critical stress value, is a boundary between elastic deformation and plastic deformation. The strength of crystalline metals is closely related to the Peierls force, i.e., the intrinsic frictional stress for dislocation motion, which was well documented^33^. For BMGs, due to the lack of crystalline defects, the yield strength of BMGs is believed to be directly associated with the cohesive strength between atomic clusters^34^,^35^,^36^. Furthermore, the yield strength is associated with the size of the elementary deformation units^30^,^37^. The increase of elastic stress causes the atomic-bond anisotropic reorientation in the first nearest-neighbor shell, and then induces surrounding atoms shift concordantly, i.e., the atomic bonds elastically stretching (or shrinking) in other atomic shells^11^,^37^,^38^. Accordingly, the elementary deformation units for delivering strain are formed. The formation of the deformation units is associated with the stress increment, i.e., the elastic energy accumulation. The stress increment and the size of the deformation unit can dominate the yield strength of BMGs^37^,^39^. At cryogenic temperature, the size of the deformation unit is enlarged due to the increases in elastic modulus and shear modulus^11^. Therefore, decreasing temperature to the cryogenic level can apparently enlarge the size of elementary deformation units^11^, which further increases the activation energy of deformation units, and results in the increase of yield strength.

Based on abundant experimental results, a constitutive model, i.e., James-Cook equation, was proposed to successfully describe the yield strength, *σ*, of metals as a function of strain rate and temperature^40^, which is expressed as,


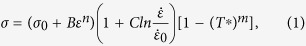


where *σ*_0_ and 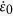 are the reference yield stress, and the reference strain rate, respectively; *ε* is the strain; *n* is the work-hardening coefficient; *B, C*, and *m* are the factors associated with the materials. T* is calculated as T* = (*T*-*T*_r_)/(*T*_m_-*T*_r_) (here *T* is the temperature, and *T*_m_ is the melting temperature. *T*_r_ is the reference temperature, at which *σ*_0_ and 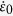 are measured). In the present study, for each MG (i.e., the Ce-MG or the Zr-MG), the *B, C*, and *m* values are constants, and the term of (σ_0_+*Bε*^*n*^) is also treated as a constant. At the setting temperatures (i.e., 77 K or 298 K), the term of [1−(T^*^)^*m*^] does not change with increasing the strain rate. Therefore, Equation (1) can be rewritten as 
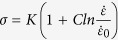
 (here *K* is a constant depending on materials), which clearly shows that the yield strength is a logarithm dependence of the strain rate. Regarding that the *C* value of metals is usually in a range from 0.007 to 0.060^41^, we assume the *C* value of 0.030 (middle value) in the present study. If the reference strain rate, 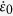, is assumed to be 10^−2^ s^−1^, the change of the yield strength of the BMGs is within 10% with decreasing the strain rate from 10^−2^ to 10^−6^ s^−1^. Due to the brittle nature of BMGs, the scattering of the yield strength value during the compression test can be already compared with the change value of the yield strength with strain rate. Thus, the yield strength of the Ce- and Zr-MGs in Fig. 2c,d do not show significantly change with strain rate. It requires to be noted that although James-Cook equation is deduced based on the mechanical properties of crystalline materials, it is originated from the stress state of materials, and the elastic energy accumulation and the release process, which has no any relationship with the structure of crystalline phase or amorphous phase^42^. Therefore, James-Cook constitutive model can be reasonable used in the present study.

**Figure 5:**
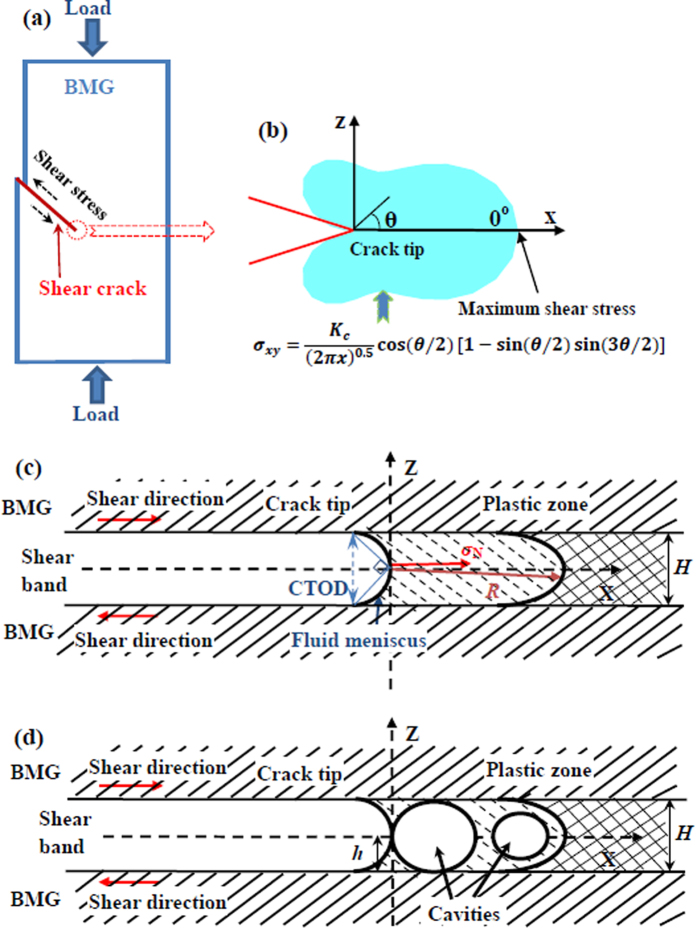
Sketch of the crack propagation process. (**a**) Sketch of shear fracture of BMGs. (**b**) Shear stress filed in the front of crack tip. (**c**) Sketch of the structure of the crack tip for BMGs. (**d**) Sketch of the cavitation formation in the plastic zone.

As demonstrated in Fig. 1, the maximum compressive strength is almost equivalent to the fracture strength of the BMGs. Therefore, the crack propagation during the fracture process may have some correlations with the compressive strength. Due to the nature of metal in BMGs, the strain can be accommodated in the atomic scale^37^. The metallic bond breaking and reformation can occur with increasing strain, accompanied with plastically softening at the atomic scale^43^. In the present study, the shear fracture of the BMGs is normally formed along a primary (main) shear band^44^. The shear fracture actually behaves a Mode II crack separation process, as shown in Fig. 5a. Based on this, it is essential to reconstructing the separation process of the crack tip during the shear fracture process, which must be associated with the fracture strength of the BMGs. The finger-like vein patterns on the fracture surface provide evidence that the locally plastic flow (softening) occurs in the crack tip during the fracture process^45^, which also suggest that the roughness on the fracture surface is characterized by peak to peak matching on two opposite fracture surfaces^46^,^47^. Therefore, locally plastic softening can effectively blunt initially sharp cracks where progressive local separation still occurs by the coalescence of the damage cavities along the extension of the shear plane.

Based on the Irwin-Orowan small-scale zone model^48^, as an in-plane shear fracture process, the crack propagation is mainly dominated by the shear stress, *σ*_*xy*_, which can be expressed as^48^,


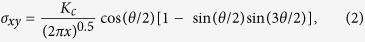


where *K*_*C*_ is the stress intensity factor, *x* is the distance to crack tip, and *θ* is the angle between the stress component and the crack propagation direction, i.e., the *X*-axis. The stress field is schematically plotted in Fig. 5b. The maximum shear stress appears when the *θ* value equals to 0°. The stress intensity, *K*_*C*_, is not a constant because the dynamic propagation causes an instable crack front, and then leads to a fluctuation in the stress intensity^49^,^50^. Therefore, we need to focus on the plastic zone in the front of crack tip.

Once the shear band is formed, due to adiabatic heating and shear dilatation, the glassy phase in shear bands is softened^51^. In this case, the crack propagation during the shear fracture process is conjectured to be a viscous fluid flowing in a channel with a height of *H*, as shown in Fig. 5c. Based on the Grease model^51^,^52^,^53^, driven by the stress, the viscous fluid in the plastic zone (in the front of crack tip) would form a fluid meniscus in the crack tip. To counter balance the surface tension of the viscous fluid, a negative stress, *σ*_N_, in the front of crack tip should be formed to pull the crack propagation along the *X*-axis^54^, which makes the fluid meniscus to be curved (Fig. 5c). The height of the fluid meniscus is defined as the crack tip open displacement (*CTOD*)^53^, which is approximately equivalent to the height of the channel, *H*, (Fig. 5c). Furthermore, the cavitation is formed in the plastic zone before the crack tip penetrating into the glassy phase. With cracking, the cavities in the plastic zone can coalesce with the main crack, and then leave the finger-like vein patterns with a height of *h* on the fracture surface. The process of the formation and coalescence of voids behave a plastic necking process when the crack tip is separated, which can leave some vein pattern on the fracture surface^47^,^55^. The height of the vein pattern, *h*, is approximated to be the half of the height of the channel, *H*^46^. Therefore, the height of the channel, *H*, is then reasonable to be treated as two times of the height of the finger-like vein pattern, *h* (Fig. 5c,d), i.e., *CTOD = H = 2h*.

**Figure 6:**
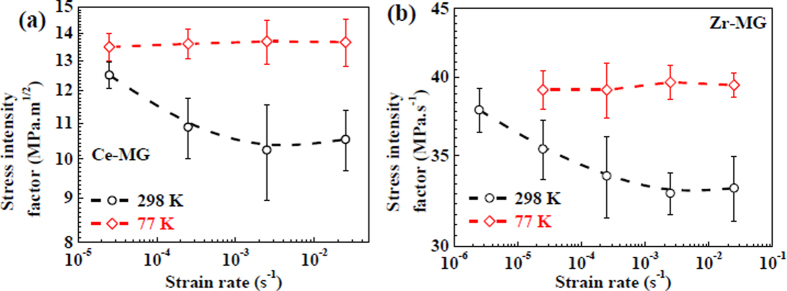
Stress intensity factors of two BMGs as functions of strain rate at different temperatures. (**a**) Ce-MG. (**b**) Zr-MG.

The *CTOD* is dominated by the stress state of the viscous layer in the front of crack tip, i.e., 
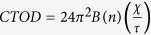
, where *χ* is the surface tension of the meniscus, *τ* is the shear stress. *B*(*n*) is a function associated with the viscosity of the viscous layer in the crack tip, the strain-rate sensitivity exponent, and the percolation wavelength of the crack front^53^,^55^. The plastic-shear resistance is a function of the equivalent shear-strain rate. For media with close to an ideal plastic behavior, the dependence of the shear resistance on the shear-strain rate is very weak, and the shear resistance may be taken as the yield strength, *σ*_*Y*_, in shear for a relatively fast laboratory compression experiment^53^. Therefore, from the fracture parameters, the critical stress intensity factor, *K*_*C*_, can be expressed as^53^,^56^


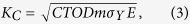


where *m* is a dimensionless constant which is determined by the material properties and the stress states, *E* is the elastic modulus, and *σ*_Y_ is the yield strength. For the fracture of amorphous solids, or the cleavage of crystalline solids, at the tip of a very sharp crack the yield strength of the solid is reached before any plastic deformation can be initiated^57^,^58^,^59^. Thus, the strength in Eq. (3) is chosen as the yield strength. The *K*_*C*_ value reflects a critical stress intensity near a crack tip caused by a remote load, which can support the crack to continuously propagate^60^. In the present study, the value of *m* is approximately 1.16 for BMGs^53^. The yield strengths of two BMGs are plotted in Fig. 2c,d. The elastic moduli of the BMGs at two temperatures have been estimated by the Varshni equation, which are listed in Table 1^61^. Accordingly, the *K*_C_ values as functions of strain rates for two BMGs at two temperatures are plotted in Fig. 6. It can be seen that the stress intensity factor of the Ce-MG at 298 K decreases from 12.5 ± 0.5 MPa·m^0.5^ to 10.2 ± 1.3 MPa·m^0.5^, and the value of the Zr-MG decreases from 37.9 ± 1.4 MPa·m^0.5^ to 33.1 ± 1.8 MPa·m^0.5^. At 77 K, the stress intensity factors of the Ce-MG and the Zr-MG at different strain rates are almost constants of 13.6 ± 0.8 MPa·m^0.5^, and 39.5 ± 1.5 MPa·m^0.5^, respectively. It can be seen that the stress intensity factor of the Ce-MG at 77 K is always larger than that 298 K. For the Zr-MG, the stress intensity factor at 77 K is also larger than the value at 298 K when the strain rate is in the range from 10^−4^ s^−1^ to 10^−2^ s^−1^.

The small-scale yielding model indicates that fracture surface separation process is confined to the frontal plastic zone^48^. The radius of the plastic zone, *R*, for plane strain can be estimated as:


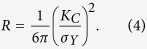
[Table t1]

Then, based on the values in Fig. 6, the size of the plastic zone, *R*, can be calculated and listed in Table 2. According to the Irwin crack tip stress field solution^48^, and the plastic zone size calculation, i.e., Eq. (2), the negative stress, *σ*_*N*_, in the plastic zone is equivalent to the shear stress component along the *X*-axis, i.e., *σ*_*xy*_ in Eq. (2). As the negative stress is along the *X*-axis, and the two fracture surfaces shear with each other, the *θ* value is 0°, and then 
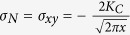
. The derivative of the *σ*_*xy*_ is the negative stress gradient, *dσ*_*N*_*/dx*. The absolute value of the negative stress gradient, │*dσ*_*N*_*/dx*│, can be expressed as,


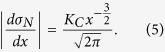


**Figure 7:**
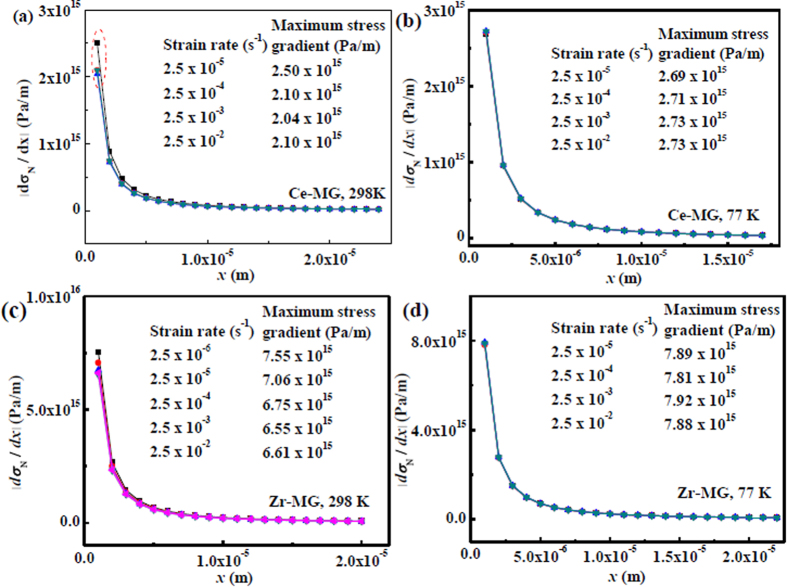
Negative pressure gradient distribution at different strain rates and temperatures. Maximum negative pressure gradient values are listed. (**a**) Ce-MG at 298 K. A stable negative pressure gradient is marked. (**b**) Ce-MG at 77 K. (**c**) Zr-MG at 298 K. (**d**) Zr-MG at 77K.

**Figure 8:**
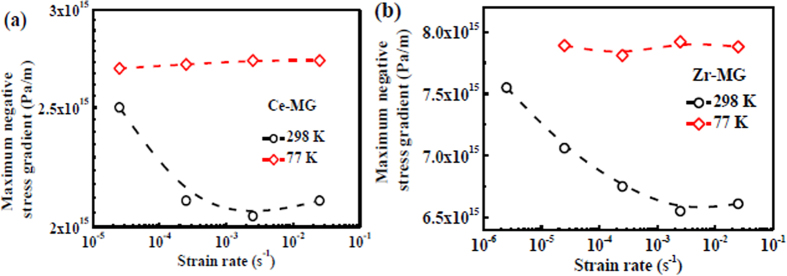
Maximum negative pressure gradients of two BMGs as functions of strain rate at different temperatures. (**a**) Ce-MG. (**b**) Zr-MG.

In the plastic zone, the distributions of negative stress gradient at different distances as well as different strain rates are plotted in Fig. 7. A dramatic decrease of the │*dσ*_N_/*dx*│ value in the plastic zone near the crack tip occurs. Thereafter, with increasing the distance to the crack tip, the negative stress gradient approaches a stable state, as shown in Fig. 7a. Because the negative stress formed in the front of crack can dominate the instability of the crack front, the │*dσ*_N_/*dx*│ value near the crack tip, i.e., the maximum negative stress gradient, is a key parameter to correlate to the crack formation and propagation. The maximum negative stress gradient vs. strain rate at different temperatures are plotted in Fig. 8, which shows that the negative pressure gradients of two BMGs decreases with increasing strain rate at 298 K, but are kept at constants at 77 K. This result is consistent with the trend of the maximum compressive strength variation. Therefore, the maximum compressive strength is assumed to be correlated with the fracture (shear cracking) behavior of BMGs. The driving force of the crack propagation in primary shear band dominates the compressive strength (fracture strength). At 77 K, the low temperature causes the rejuvenation of the glassy phase^62^. In this case, the activation energy of the locally softening in the front of crack tip, i.e., in the plastic zone, is more difficult than that at 298 K^62^. Thus, the negative stress gradient is improved, which further enhances the compressive strength. Furthermore, at cryogenic temperature, the thermal diffusion is enhanced due to the enlarged temperature gradient^63^. The effect of adiabatic heating is weakened, which possibly cause that the viscosity of the viscous fluid, i.e., the softened glassy phase, in shear bands significantly increases. In this case, the negative pressure gradient is mainly influenced by temperature rather than strain rate. Thus, the maximum compressive strength is constant at 77 K with increasing strain rate.

## Conclusions

In summary, we profile the mechanical responses of two BMGs deformed at different strain rates and temperatures. The yield strength and the maximum compressive strength (i.e., fracture strength) are chosen as the indexes reflecting the deformation behavior of the BMGs. The yield strength is found to be independent of the strain rate, which is associated with the elastic energy accumulation, and the activation of the elementary deformation unit. This process is insignificantly influenced by the strain rate. The temperature at cryogenic level can enlarge the size of elementary deformation unit, and then improve the activation energy. Therefore, the yield strength is increased by decreasing the temperature. The maximum compressive strength is almost equivalent to the fracture strength that is depending on the driving force of the crack propagation, i.e., the negative stress gradient. As a dynamic process, the crack tip instability is sensitive to the strain rate, which is evidenced by the analysis of the fractographies of the BMGs fractured at different strain rates and temperatures. Our findings construct a bridge to link the strength of BMGs at the macroscale and the shear cracking behavior at the micro scale, which not only help to understand of the strength origination of BMGs but may also provide fundamental insights into the explanation of their unique mechanical behavior.

## Methods

The Zr_52.5_Cu_17.9_Ni_14.6_Al_10_Ti_5_ and Ce_68_Al_10_Cu_20_Co_2_ alloys were prepared by arc melting a mixture of pure metal elements (with purities higher than 99.99%) in an argon atmosphere, followed by suction casting into Cu-mould to form rod-like BMGs. The diameter was 2 mm, and the length was 70 mm for the BMGs. The glassy phase of each as-cast BMG was examined by X-ray diffraction (XRD) technique in a Rigaku DLMAX-2550 diffractometer with the Cu-K_α_ radiation (not shown). The fractographies of the fractured BMGs were observed in an APHENOM^TM^ G2 (FEI company) scanning electron microscope (SEM). Compression specimens with a length/diameter ratio of 2 were cut from the rod-like BMGs. Two parallel ends of each specimen were carefully ground to that the surface roughness was less than 1 μm. Compression tests were conducted using a MTS CMT5205 machine with a strain rate ranging from 2.5 × 10^−6^ s^−1^ to 2.5 × 10^−2^ s^−1^, at 298 K and 77 K, respectively. To exclude the occasional case, the compression tests of these two BMGs were repeated at least four times at each temperature and each strain rate.

## Additional Information

**How to cite this article**: Wang, Y.W. *et al*. Rate dependent of strength in metallic glasses at different temperatures. *Sci. Rep*. **6**, 27747; doi: 10.1038/srep27747 (2016).

## Supplementary Material

Supplementary Information

## Figures and Tables

**Table 1 t1:** Elastic moduli of two BMGs at different temperatures.

BMGs	*T* (K)	*E* (GPa)
Ce-MG	298	31.4
Ce-MG	77	33.9
Zr-MG	298	88.6
Zr-MG	77	90.2

**Table 2:** Stress intensity factor and size of plastic zone of two BMGs.

BMGs	*T* (K)	Strain rate (s^−1^)	*K*_C_(MPa·m^0.5^)	*R* (μm)
Ce-MG	298	2.5 × 10^−5^	12.5 ± 0.5	36.2 ± 3.0
Ce-MG	298	2.5 × 10^−4^	10.5 ± 0.8	25.4 ± 4.4
Ce-MG	298	2.5 × 10^−3^	10.2 ± 1.3	24.1 ± 7.4
Ce-MG	298	2.5 × 10^−2^	10.5 ± 0.9	25.9 ± 4.7
Ce-MG	77	2.5 × 10^−5^	13.5 ± 0.5	17.3 ± 1.5
Ce-MG	77	2.5 × 10^−4^	13.6 ± 0.5	17.9 ± 1.7
Ce-MG	77	2.5 × 10^−3^	13.7 ± 0.8	18.1 ± 2.4
Ce-MG	77	2.5 × 10^−2^	13.7 ± 0.9	17.8 ± 2.3
Zr-MG	298	2.5 × 10^−6^	37.9 ± 1.4	27.3 ± 3.0
Zr-MG	298	2.5 × 10^−5^	35.4 ± 1.8	23.6 ± 2.7
Zr-MG	298	2.5 × 10^−4^	33.8 ± 2.3	21.6 ± 3.1
Zr-MG	298	2.5 × 10^−3^	32.8 ± 1.2	20.4 ± 2.0
Zr-MG	298	2.5 × 10^−2^	33.1 ± 1.8	20.8 ± 2.4
Zr-MG	77	2.5 × 10^−5^	39.5 ± 1.3	22.7 ± 1.6
Zr-MG	77	2.5 × 10^−4^	39.2 ± 1.8	22.1 ± 2.2
Zr-MG	77	2.5 × 10^−3^	39.7 ± 1.2	22.9 ± 1.7
Zr-MG	77	2.5 × 10^−2^	39.5 ± 0.8	22.6 ± 1.5
