# Supplementary material for: Rate dependent of strength in metallic glasses at different temperatures
Source: Sci Rep. 2016 Jun 8;6:27747. doi: 10.1038/srep27747 (PMC4897709; doi:10.1038/srep27747)
Supplement: Supplementary Information [file srep27747-s1.pdf]

Supplementary Information

Rate dependent of strength in metallic glasses at different temperatures

Y.W. Wang, X.L. Bian, S.W. Wu, I. Hussain, Y.D. Jia, J. Yi, G. Wang*

Laboratory for Microstructures, Institute of Materials, Shanghai University, Shanghai 200444, China

*Corresponding author: g.wang@shu.edu.cn

I. Summary of nominal stress-displacement curves of two BMGs

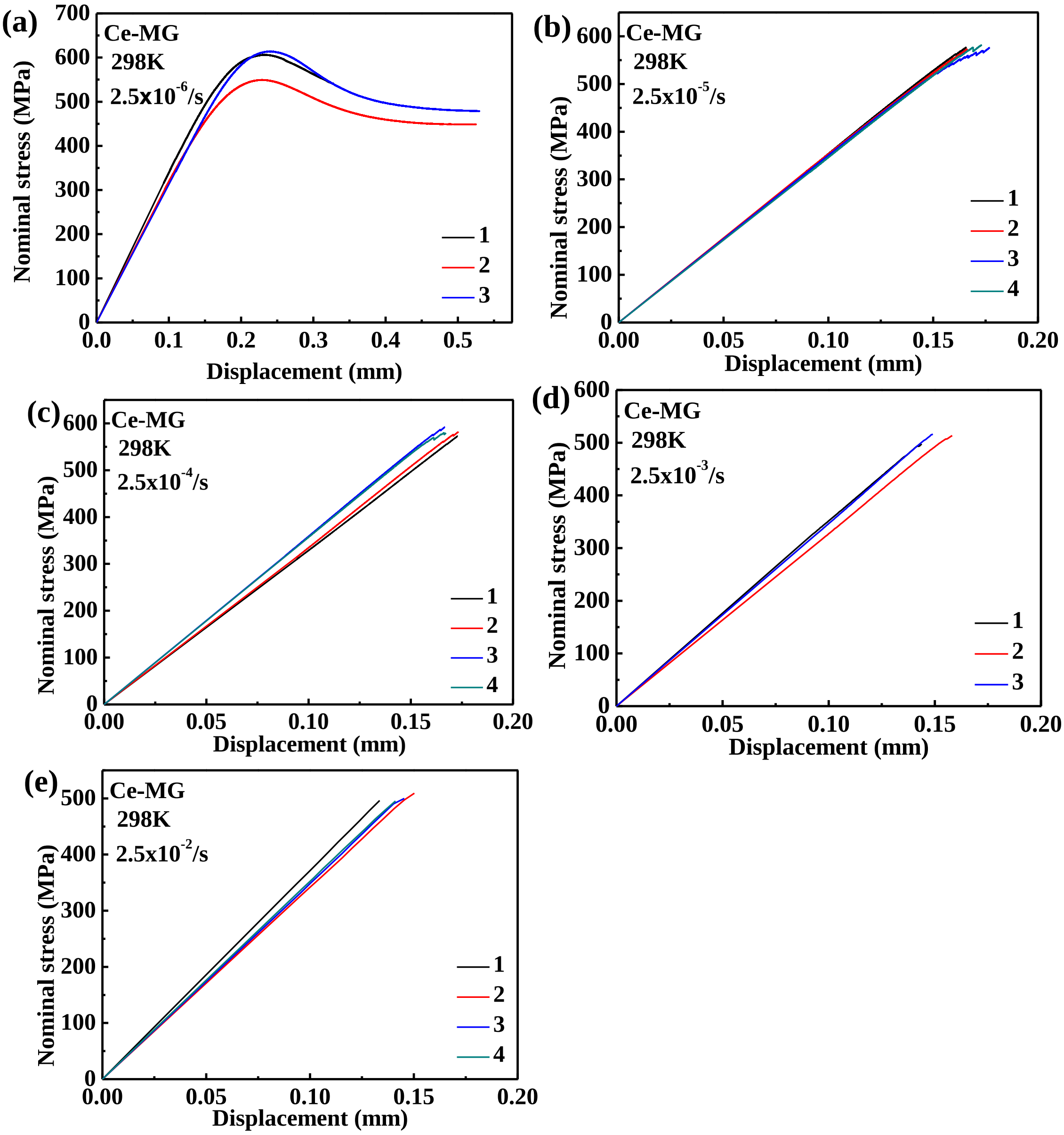

Fig. S1 Nominal stress-displacement curves of Ce-MG at 298 K.

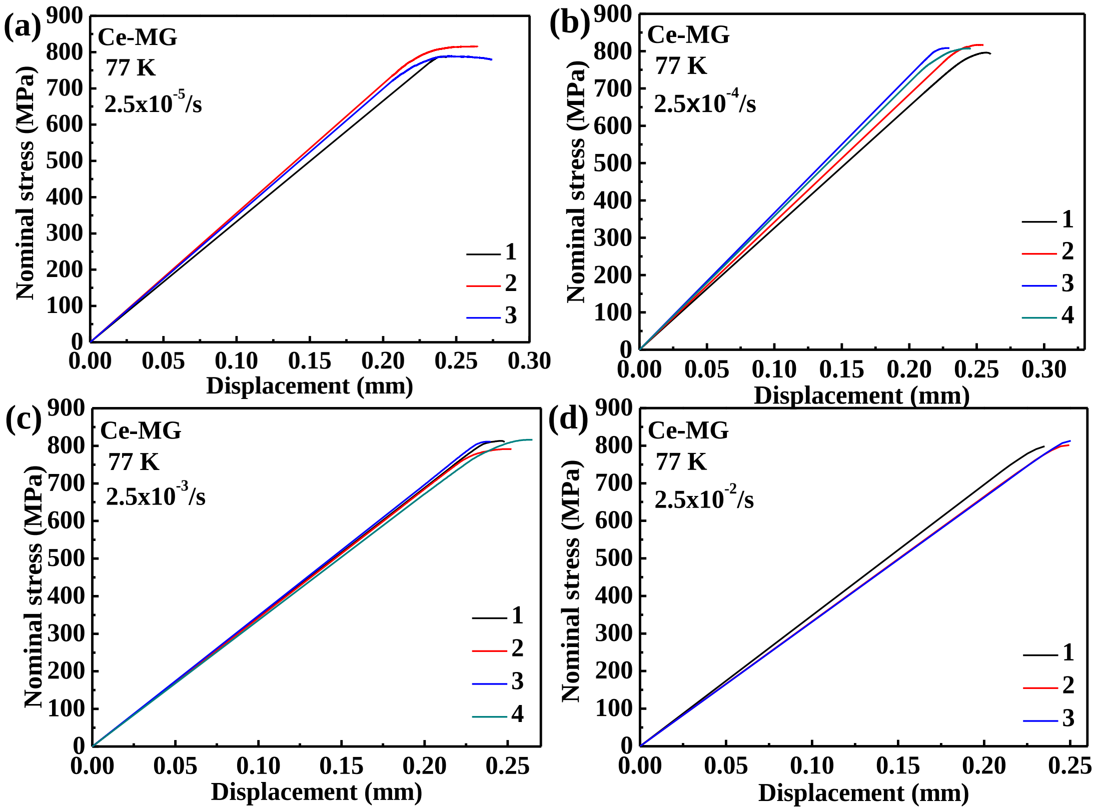

Fig. S2 Nominal stress-displacement curves of Ce-MG at 77 K.

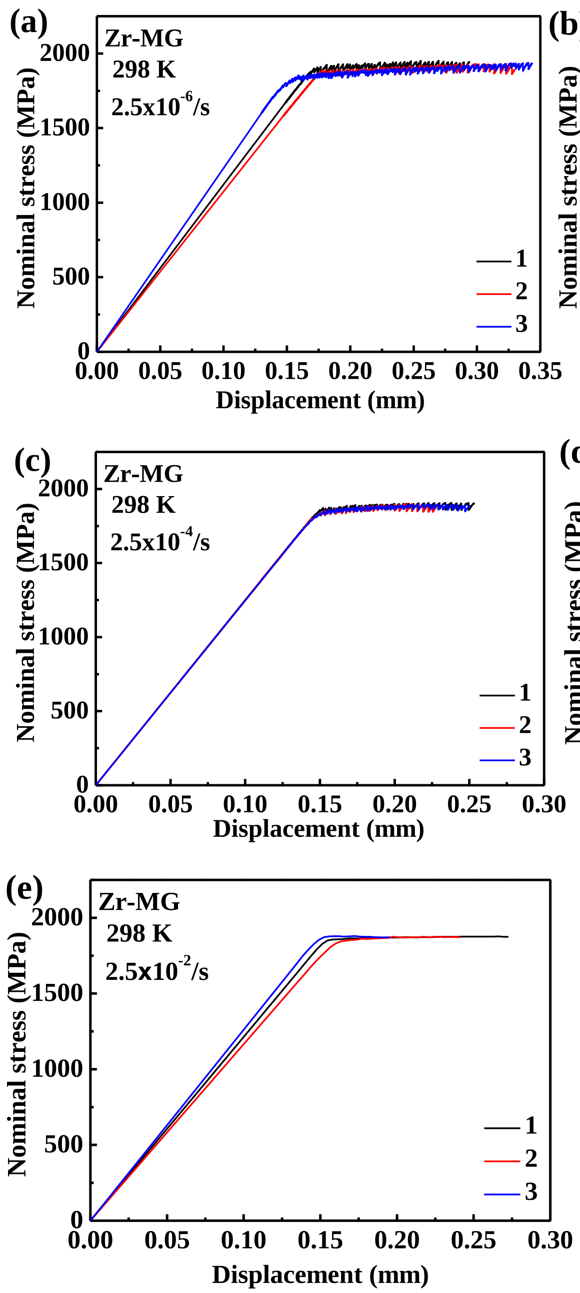

Fig. S3 Nominal stress-displacement curves of Zr-MG at 298 K.

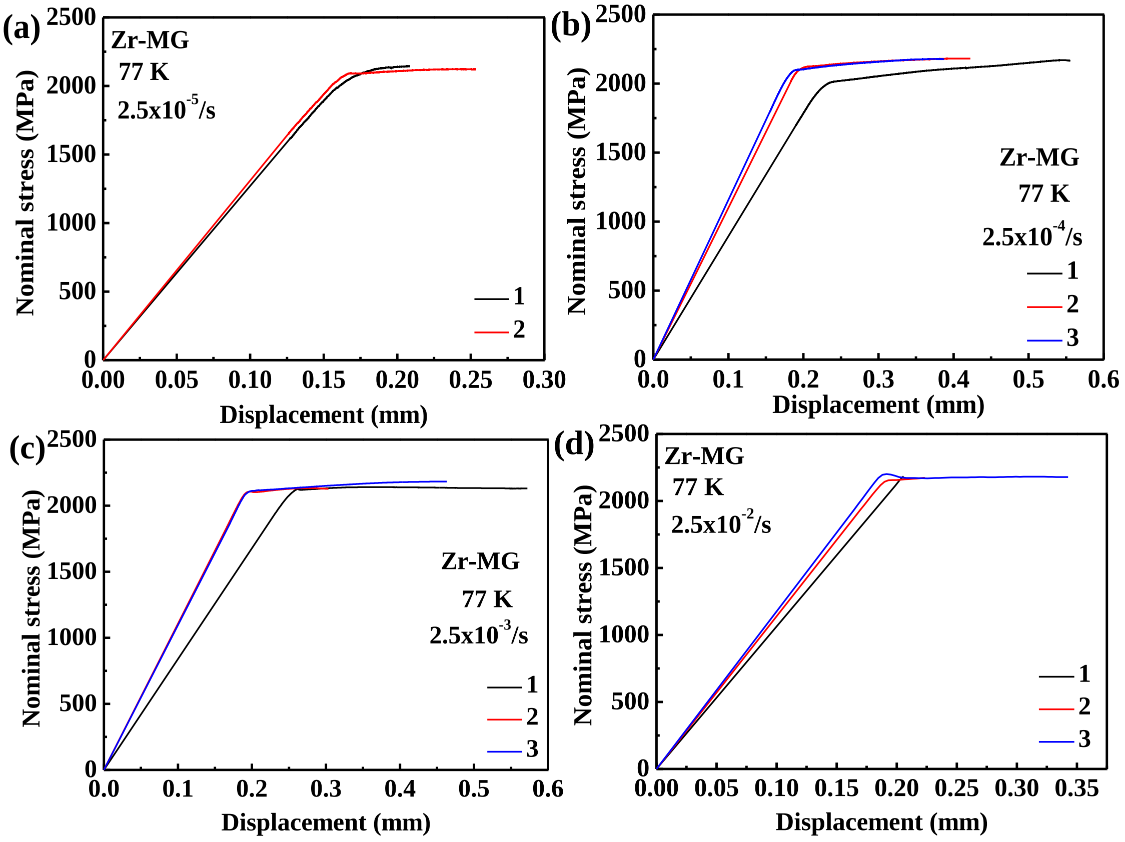

Fig. S4 Nominal stress-displacement curves of Zr-MG at 77 K.

II. Determination of the height of the fingerlike pattern

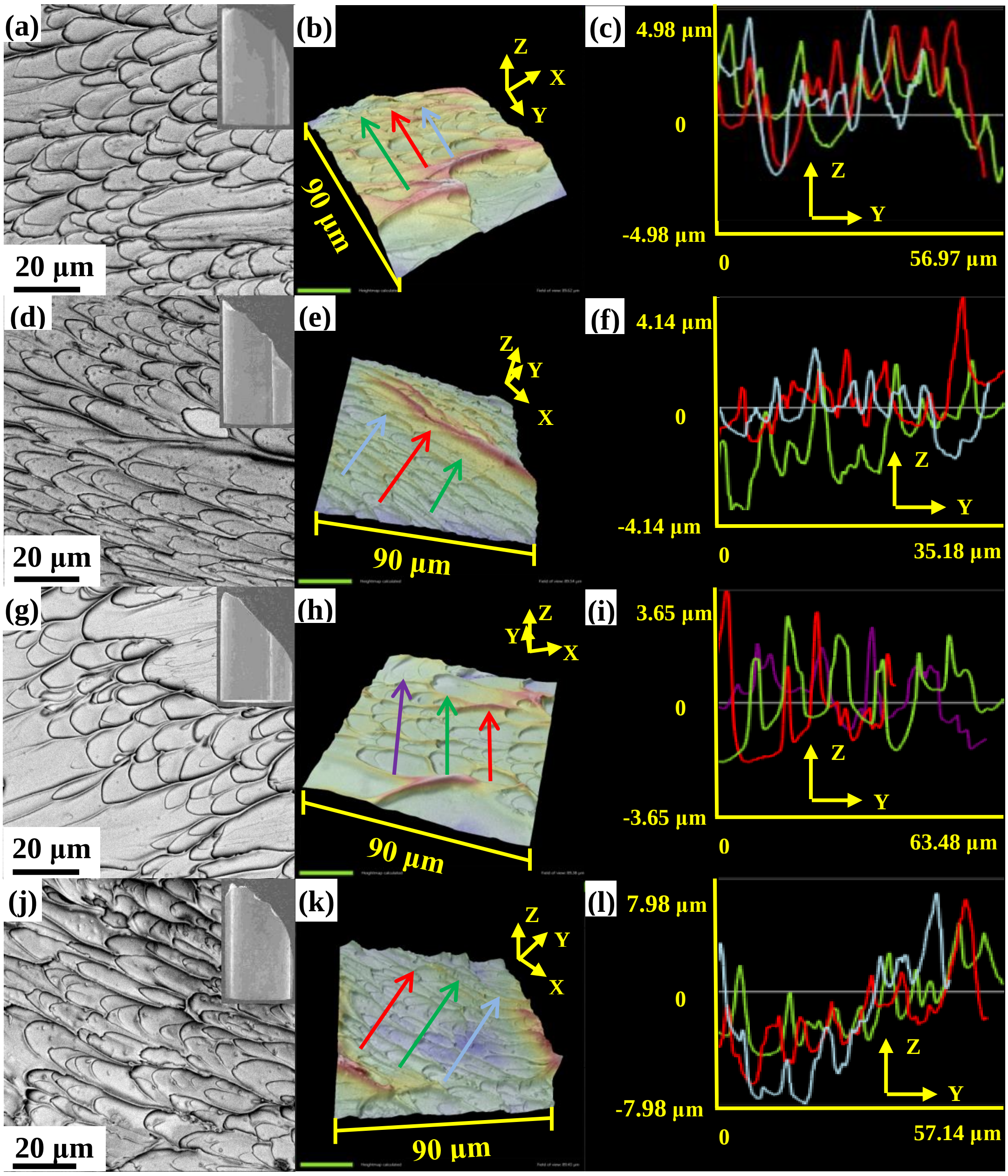

Fig. S5 Fracture morphologies of Ce-MG fractured at 298 K. (a) Fractography at 2.5×10^{-5} s^{-1}. (b) 3D fractographies at 2.5×10^{-5} s^{-1}. (c) Three sectional shapes reflecting the surface roughness along the three lines in (b). (d) Fractography at 2.5×10^{-4} s^{-1}. (e) 3D fractographies at 2.5×10^{-4} s^{-1}. (f) Three sectional shapes reflecting the surface roughness along the three lines in (e). (g) Fractography at 2.5×10^{-3} s^{-1}. (h) 3D fractographies at 2.5×10^{-3} s^{-1}. (i) Three sectional shapes reflecting the surface roughness along the three lines in (h). (j) Fractography at 2.5×10^{-2} s^{-1}. (k) 3D fractographies at 2.5×10^{-2} s^{-1}. (l) Three sectional shapes reflecting the surface roughness along the three lines in (k).

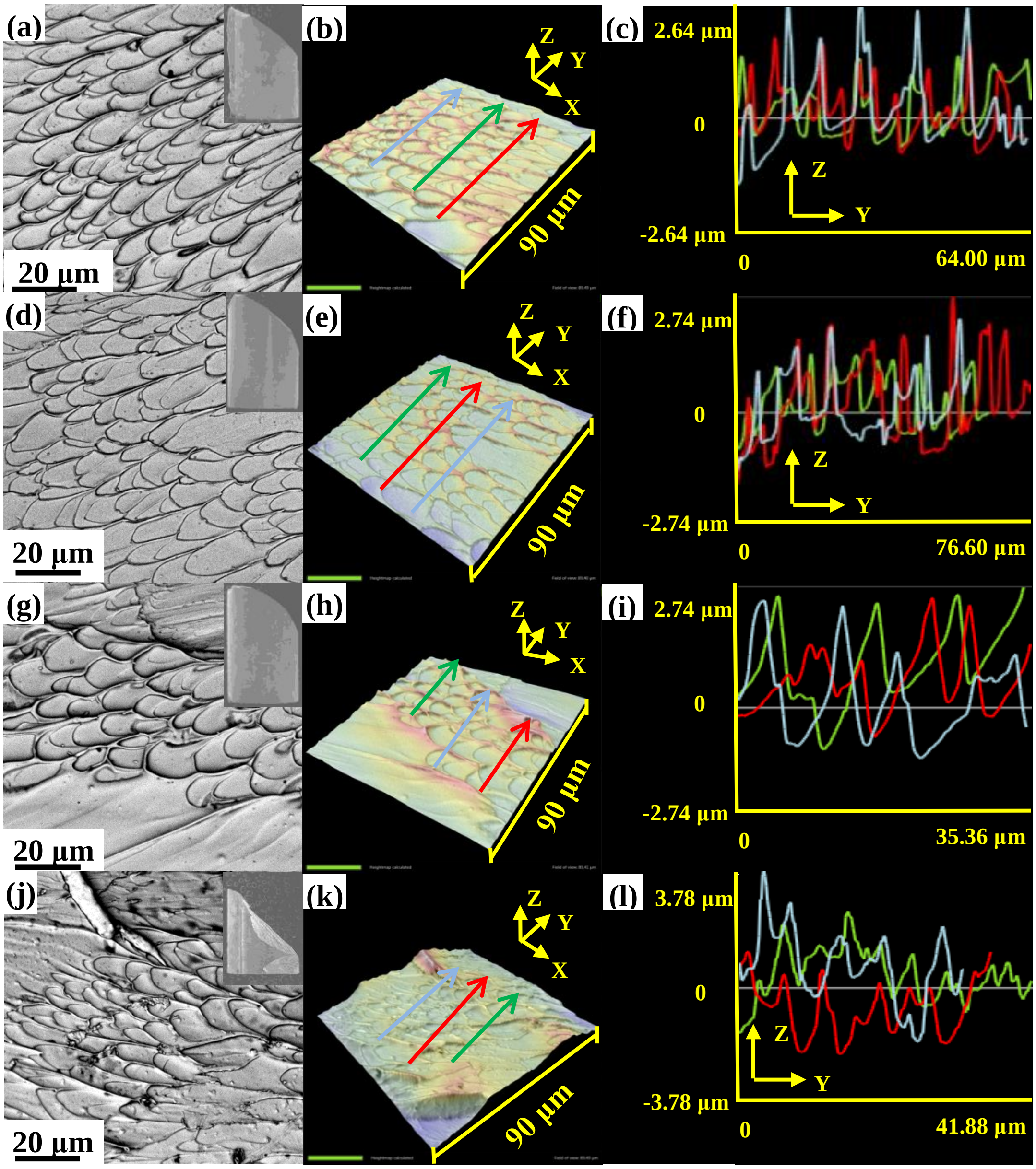

Fig. S6 Fracture morphologies of Ce-MG fractured at 77 K. (a) Fractography at 2.5×10^{-5} s^{-1}. (b) 3D fractographies at 2.5×10^{-5} s^{-1}. (c) Three sectional shapes reflecting the surface roughness along the three lines in (b). (d) Fractography at 2.5×10^{-4} s^{-1}. (e) 3D fractographies at 2.5×10^{-4} s^{-1}. (f) Three sectional shapes reflecting the surface roughness along the three lines in (e). (g) Fractography at 2.5×10^{-3} s^{-1}. (h) 3D fractographies at 2.5×10^{-3} s^{-1}. (i) Three sectional shapes reflecting the surface roughness along the three lines in (h). (j) Fractography at 2.5×10^{-2} s^{-1}. (k) 3D fractographies at 2.5×10^{-2} s^{-1}. (l) Three sectional shapes reflecting the surface roughness along the three lines in (k).

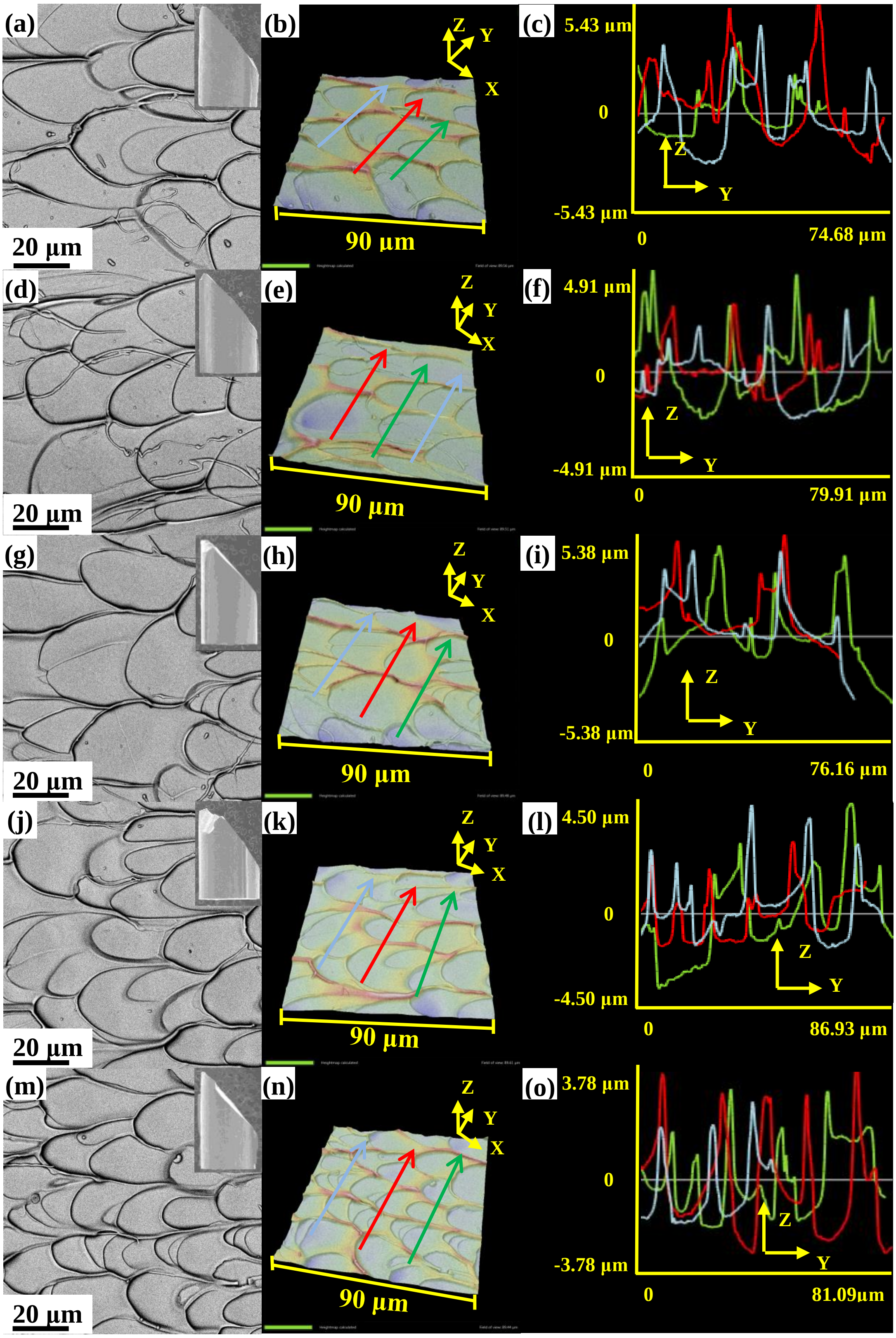

Fig. S7 Fracture morphologies of Zr-MG fractured at 298 K. (a) Fractography at 2.5×10^{-6} s^{-1}. (b) 3D fractographies at 2.5×10^{-6} s^{-1}. (c) Three sectional shapes reflecting the surface roughness along the three lines in (b). (d) Fractography at 2.5×10^{-5} s^{-1}. (e) 3D fractographies at 2.5×10^{-5} s^{-1}. (f) Three sectional shapes

reflecting the surface roughness along the three lines in (e). (g) Fractography at 2.5×10^{-4} s^{-1}. (h) 3D fractographies at 2.5×10^{-4} s^{-1}. (i) Three sectional shapes reflecting the surface roughness along the three lines in (h). (j) Fractography at 2.5×10^{-3} s^{-1}. (k) 3D fractographies at 2.5×10^{-3} s^{-1}. (l) Three sectional shapes reflecting the surface roughness along the three lines in (k). (m) Fractography at 2.5×10^{-2} s^{-1}. (n) 3D fractographies at 2.5×10^{-2} s^{-1}. (o) Three sectional shapes reflecting the surface roughness along the three lines in (n).

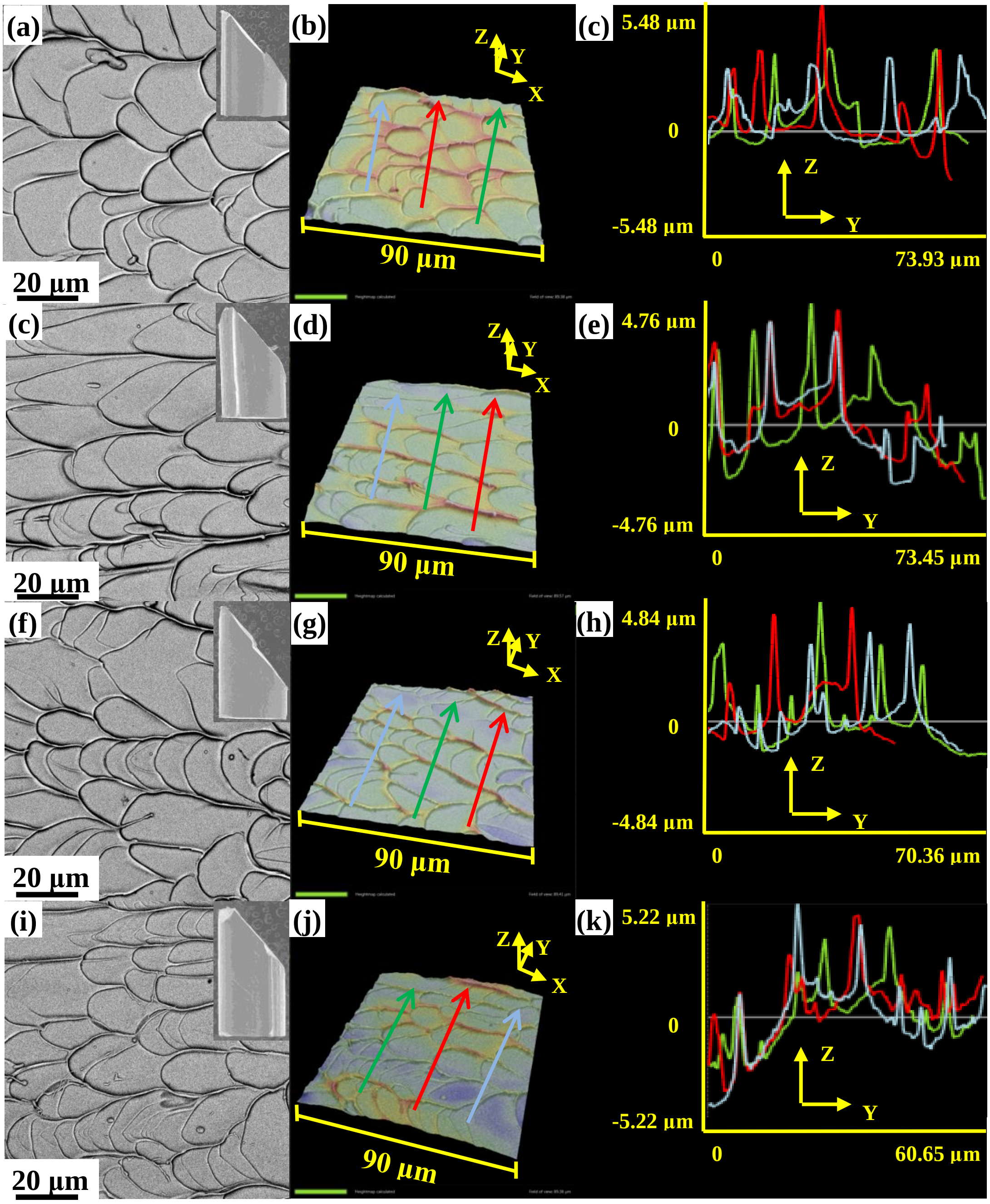

Fig. S8 Fracture morphologies of Zr-MG fractured at 77 K. (a) Fractography at 2.5×10^{-5} s^{-1}. (b) 3D fractographies at 2.5×10^{-5} s^{-1}. (c) Three sectional shapes reflecting the surface roughness along the three lines in (b). (d) Fractography at 2.5×10^{-4} s^{-1}. (e) 3D fractographies at 2.5×10^{-4} s^{-1}. (f) Three sectional shapes reflecting the surface roughness along the three lines in (e). (g) Fractography at 2.5×10^{-3} s^{-1}. (h) 3D fractographies at 2.5×10^{-3} s^{-1}. (i) Three sectional shapes reflecting the surface roughness along the three lines in (h). (j) Fractography at 2.5×10^{-2} s^{-1}. (k) 3D fractographies at 2.5×10^{-2} s^{-1}. (l) Three sectional shapes reflecting the surface roughness along the three lines in (k).
